# Blueberry Supplementation in Neuronal Health and Protective Technologies for Efficient Delivery of Blueberry Anthocyanins

**DOI:** 10.3390/biom11010102

**Published:** 2021-01-14

**Authors:** Phuong H.L. Tran, Thao T.D. Tran

**Affiliations:** 1 Deakin University, School of Medicine, IMPACT, Institute for Innovation in Physical and Mental Health and Clinical Translation, Geelong, Australia; phuong.tran1@deakin.edu.au; 2 Institute of Research and Development, Duy Tan University, Danang 550000, Vietnam; 3 The Faculty of Pharmacy, Duy Tan University, Danang 550000, Vietnam

**Keywords:** blueberries, anthocyanins, neuronal health, bioavailability, supplementation forms, protective technologies

## Abstract

Blueberries are consumed as healthy fruits that provide a variety of benefits to the nervous system. Scientists have found that blueberries can be used as a daily edible source for supplementation to prevent and minimize complexities of age-related diseases as well as to improve learning and memory in children. Anthocyanins are the most mentioned compounds among the components in blueberries, as they play a major role in providing the health benefits of this fruit. However, while they are highly active in impeding biological impairment in neuronal functions, they have poor bioavailability. This review focuses on neurological investigations of blueberries from in vitro cell studies to in vivo studies, including animal and human studies, with respect to their positive outcomes of neuroprotection and intervention in neurodegenerative conditions. Readers will also find information on the bioavailability of anthocyanins and the considerable factors affecting them so that they can make informed decisions regarding the daily consumption of blueberries. In this context, the ways in which blueberries or blueberry supplementation forms are consumed and which of these forms is best for maximizing the health benefits of blueberries should be considered important decision-making factors in the consumption of blueberries; all of these aspects are covered in this review. Finally, we discuss recent technologies that have been employed to improve the bioavailability of blueberry anthocyanins in the development of effective delivery vehicles supporting brain health.

## 1. Introduction

It has been well established that a rich diet of fruits and vegetables yields sustainable health benefits and reduces risks of lifestyle-associated health conditions. Blueberries (BBs) are one of the fastest growing fresh produce categories due to the high demand of customers [1], who have demonstrated an increased awareness of BBs’ benefits to human health. Interestingly, BBs have been labeled a “super fruit” or “superfood” for their ability to prevent or mitigate several diseases, such as cardiovascular diseases, diabetes, and cancer [2,3,4]. Owing to the rich source of polyphenols (such as anthocyanins (ACNs), flavanols, and pterostilbene), BBs display a variety of biological activities in vitro and in vivo for the prevention of carcinogenesis, including the inhibition of the production of pro-inflammatory molecules, oxidative stress, and the products of oxidative stress, such as DNA damage (with 400 µg/mL ACN in rat models), cancer cell proliferation (with 40 µg/mL pterostilbene in rat models), and an increase in apoptosis (with 50–150 µg/mL ACN) [5,6]. The consumption of 250 g of BBs per day for 6 weeks and 375 g of BBs 1 h to 2.5 h prior to running in a study investigating the effects of BBs on exercise activities demonstrated promising results, including increased natural killer cell counts, reduced oxidative stress, and increased anti-inflammatory cytokines [7]. Szeto et al. recently performed an in vivo study with human leucocytes to evaluate the ability of both BB juice and fruit to protect leucocyte DNA against exogenous oxidative stress [8]. They showed that a single dose of 200 mL BB juice lowered DNA damage by 19%, and remarkably, a 40 g BB fruit supplement decreased cellular DNA damage by 28% within 2 h [8]. In another study, volunteers over forty years of age with a body mass index of approximately 25 kg/m^2^ received a drink containing 25 g freeze-dried BB powder or a placebo drink, and the BB group showed that consumption for 6 weeks significantly reduced the levels of endogenously oxidized DNA bases and increased resistance to H_2_O_2_-induced DNA damage [9].

In addition, BBs are beneficial in the treatment of age-related diseases, as they have been reported to alleviate behavioral deficits in ageing animals consuming a high-fat diet [10]. Neurodegenerative diseases such as Parkinson’s disease (PD), Alzheimer’s disease (AD), ischemic diseases, etc., are associated with ageing and are growing concerns with very few treatments. Scientists have found that the regular intake of polyphenol-rich fruit such as BBs can delay the onset of brain ageing and neurodegenerative disorders due to their strong antioxidative and anti-inflammatory properties [11,12,13]. The brain is likely to suffer from oxidative stress more than other organs, as its antioxidant defence systems are susceptible to low activity [14]. Because of the elevated release of reactive oxygen species (ROS), scientists have also identified the important role of neuroinflammatory processes in the brain in the development of neurodegenerative disorders [15,16]. A decrease in age-related neuronal and behavioral indices resulting from oxidative stress may be relieved by antioxidants. The abundance of antioxidants in BBs has been shown to boost brain performance and improve memory in all ages and enhance and protect brains in older adults, as the flavanols in BBs interact directly with neurons at the molecular level, initiating signaling pathways that increase connections between neurons, communications between cells, and stimulate neuronal regeneration. Hence, the consumption of BBs should be part of a daily healthy eating lifestyle. In this context, one concern may be raised: in what form should BBs consumed to obtain the best health benefits? BB juice, freshly picked/frozen BBs, or BBs incorporated into a variety of recipes are common forms of consumption, but it remains to be seen which recipe would be the best one to achieve the highest beneficial effects on brain health. The total content of polyphenols in BBs accounts for 48–304 mg out of 100 g of whole fruit, whereas ACNs account for 25 to 495 mg for every 100 g of BB [17,18]. There are different types of ACNs in BBs, namely, malvidin, delphinidin, petunidin, cyanidin, and peonidin [19]. The phenyl-benzopyrylium skeleton is the fundamental structure of ACNs, which bear hydroxy (-OH) and/or methoxy (-OCH_3_) groups [20]. The differences in position and number of these groups result in different ACNs and their antioxidant properties [20]. The content of ACNs is maintained in overripened BBs and may increase after storage [21]. One of the most common transformations of whole fruit BBs for consumption is BB juice. The loss of ACNs during the process of juicing should be considered. Although 85% of total monomeric anthocyanins could be maintained in BB juice [22], up to 55% of ACNs could be lost after the juicing process [23]. Thus, a suitable process for obtaining the best health benefit from BBs should be thoroughly considered and evaluated. Furthermore, to achieve the best desired pharmacological action, administration at the most dissolved state in the aqueous milieu of the gastrointestinal (GI) tract is ideal for permeating the GI tract membranes to enter systemic circulation and hence reach the greatest absorption. No single factor is responsible for efficiently delivering an active agent to the brain through the blood-brain barrier; rather, several complex measures should be considered, including the hydrophobicity of the substance with respect to the lipophilic barrier, the permittivity of the barrier itself, enzymes in the brain, and the surface activity and relative size of the reagent.

The most widely known compounds that produce the health benefits of BBs are ACNs, the bioactive compounds that give BBs a blue colour. Unexpectedly, the in vivo bioactivity of ACNs has shown controversial results or been explicitly found to not have effects as great as they have been recorded in in vitro tests [24,25,26]. The reasons for this result are the instability of ACNs in the GI tract and their low bioavailability. Although these results came from vision and eye health studies and have not been demonstrated in neurological studies of in vitro and in vivo tests where researchers could achieve similar effects of ACNs, the reality of the poor bioavailability of ACNs threatens the advantages of BB daily intake. This review will hence show readers a picture of BBs’ valuable biological activity in terms of their effects on brain health, such as neuroprotection and intervention in neurological disorders, from in vitro to in vivo studies, including typical human studies on BB interventions. While mainly providing information on ACNs, concomitantly, the review will present factors affecting the bioavailability of ACNs, choices of BB intake form and recent technologies used to protect ACNs, all to maximize the benefits of BBs in brain health.

## 2. Bioactivity of BBs on Brain Health Protection—From In Vitro to Animal Studies

BBs consist of enriched bioactive compounds, including ACNs and other polyphenols, phenolic acids, and stilbene derivatives, that have demonstrated significant antioxidant and anti-inflammatory properties [27,28]. Many neurodegenerative conditions are involved in neuroinflammation (Table 1) [29].

**Figure 1:**
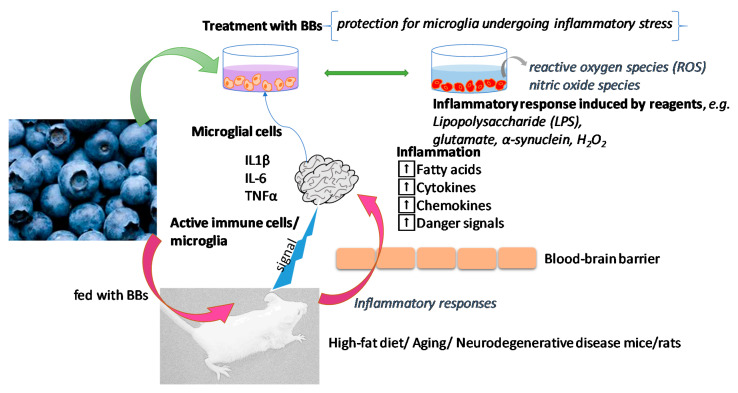
Effects of blueberry (BB) supplementation on brain dysfunction in in vitro and animal models. BBs can reduce the activation of microglial cells and decrease the release of ROS and/or nitric oxide species produced by inflammatory responses induced by reagents in in vitro studies. Meanwhile, BBs also demonstrate their neuroprotective effects in high-fat diet/ageing/neurodegenerative disease animal models against inflammatory responses regulated by peripheral pro-inflammatory molecules (fatty acids, cytokines, chemokines, danger signals), which can signal the immune cells of the brain (mainly represented by microglia) to release pro-inflammatory molecules such as interleukin-1beta (IL1β), IL-6, and tumor necrosis factor alpha (TNFα).

Wang et al. reported the potential anti-inflammatory effects of BBs through the NLRP3 inflammasome, which is a multi-protein complex that triggers the release of the pro-inflammatory cytokines IL-1β and IL-18 [30]. Mononuclear macrophages (RAW264.7) were used to study the effects of BB extracts on anti-inflammatory activity. Lipopolysaccharides (LPSs) have been used to activate RAW264.7 cells to produce ROS in mitochondria and induce mitochondrial dysfunction to further stimulate the NLRP3 inflammasome, i.e., the gene expression of _Mus_IL-1b, _Mus_NLRP3, _Mus_Caspase-1, _Mus_ASC, _Mus_TNF-a, _Mus_IL-6, and _Mus_iNOS and the protein expression of NLRP3 and Caspase-1 [30]. The BB extracts significantly lowered the expression of these genes and proteins [30]. In addition, increased α-synuclein clusters and excessive neurotransmitter glutamate are found in the brain in some specific neurodegenerative diseases [36,37]. Debnath-Canning et al. used microglia, the immune cells of the brain, to investigate the effects of BB fruit or leaf extracts on inflammatory responses after the cells were exposed to glutamate (100 µM) or α-synuclein (100 ng/mL) for 24 h [31]. The treatment (1 µL of sterile filtered 250 mg/mL BB fruit extract or 25 mg/mL BB leaf extract) inhibited cell death and decreased inflammatory conditions, suggesting protection by supplementation against neuroinflammation-associated neurodegenerative disorders [31]. The results of this study were also in accordance with those of another study where brain cells from rats were exposed to a high glutamate content (100 μM) for 24 h and BB fruit or leaf extracts showed their excellent ability to protect the cells from glutamate toxicity [32]. In a study by Ma et al., BB ACN-enriched extract (100 μg/mL) showed free radical scavenging, antiglycation effects and reactive carbonyl species trapping abilities that were superior to ACN-free extract, whereas BB ACN-enriched extract (20 μg/mL) decreased H_2_O_2_-induced ROS production, LPS-induced nitric oxide species, H_2_O_2_-induced cytotoxicity, and caspase-3/7 activity in microglia [33]. These results indicate the protective ability of BB ACNs against inflammatory stress in microglia. Carey et al. also demonstrated the effects of ACNs and other compounds of the BBs, including pterostilbene and resveratrol, in protecting microglia from inflammation-induced stress signaling [34]. The treatment of cells with BB extract before exposure to LPS resulted in reductions in nitric oxide and tumor necrosis factor alpha (TNFα) release and in the expression of nitric oxide synthase and cyclooxygenase-2. The research group suggested using 1 mg/mL BB extract to maximize the effects [34]. The level of ROS is controlled by antioxidant mechanisms but may become overwhelming during oxidative stress periods. Based on the possible activity of oxidative stress components and inflammatory mediators in vascular disorders, Youdim et al. investigated the antioxidant and anti-inflammatory effects of BB ACNs on human microvascular endothelial cells and found that cells that were pre-supplemented with 0.1 mg/mL ACNs were protected from TNFα-induced damage [35]. This outcome was confirmed in later reports from other research groups [38,39,40,41]. When researchers performed in vivo studies (Table 2), rats that were fed 2% BB supplementation showed a decrease in the activation of microglia, noticeably in aged host brains, implying the potential of BBs to attenuate age-induced inflammation [42]. They also found that OX-6 immunoreactive microglial cell activation in the hippocampus and immunoreactivity for the pro-inflammatory cytokine IL-6 were reduced in rats treated with BBs [42]. These study outcomes suggest the benefit of the transplantation of central nervous tissue, which is a known therapeutic intervention for age-related neurodegenerative diseases and stroke, as survival of embryonic neuronal cells can lead to the risk of circulating inflammatory cytokines and oxidative stress in the aged host brain [42]. Figure 1 demonstrates typical examples of in vitro cells and animal models employed to investigate the bioactivity of BBs against inflammatory responses in the brain.

An earlier study on 344 19-month-old rats eating BBs (18.6 g of dried aqueous extract per kilogram of diet) for 8 weeks showed the effectiveness of BBs in reversing age-related deficits in several neuronal signaling pathways and behaviors [43]. ACNs in the BBs are capable of crossing the blood-brain barrier and then locating in different brain regions associated with cognitive performance (learning and memory) [50]. Selective vulnerability to oxidative stress has been used as an important measure to determine different regions suffering from neuronal ageing-based functional decline [51,52]. As memory decline is associated with reduced hippocampal neurogenesis in elderly individuals, Casadesus et al. studied the changes in hippocampal neurogenesis, extracellular receptor kinase activation, and insulin-like growth factor-1 (IGF-1) and IGF-1R levels in aged rats fed BBs [44]. These hippocampal plasticity parameters were increased, and notably, the IGF-1 and IGF-1R levels corresponded with improved spatial memory. In addition, scientists found that there is an association between Ca^2+^ buffering deficiency and oxidative stress through the loss of Ca^2+^ recovery time in M1, M2, and M4 acetylcholine (ACh) receptors. The oxidative stress-induced loss of Ca^2+^ recovery time was determined by cell treatment with dopamine for 4 h, and BB demonstrated its effect on antagonizing this oxidative stress in M1-transfected cells treated with BB extract by decreasing the pattern of cyclic AMP response element binding protein (CREB) activation and likely decreasing the pattern of dopamine-induced protein kinase gamma (PKCgamma) [53]. BBs were also investigated for their anti-inflammatory activity in post-traumatic stress disorder (PTSD) by feeding rats a BB-enriched diet (2%) [45]. The levels of 5-hydroxytryptamine (5-HT) and norepinephrine (NE), biogenic amines that act as the principal neurotransmitters for fast excitatory synaptic responses in the central nervous system and have other impacts on different brain regions susceptible to stress responses [54], were modulated (5-HT levels but not NE levels were increased). BBs were shown to diminish oxidative stress, decrease the levels of inflammatory cytokines, and reimpose neurotransmitter balance in a PTSD rat model [45]. Recently, based on the fact that a high-fat diet (HFD) may lead to dysfunction of the central nervous system, including increased oxidative stress in the brain and reduced neuroplasticity, similar to age-related complex issues, and from reports on the effects of BBs on neuroprotection, HFD mouse models were fed freeze-dried BBs (4%), and their brain tissue was assessed to determine the neuroprotective mechanisms responsible for ameliorating cognitive dysfunction associated with HFD [46]. This study showed that BB consumption resulted in fewer microglia and increased neuroplasticity in the brains of HFD mice than in those of HFD mice who did not consume BBs and of low-fat diet mice [46]. On the other hand, Maulik et al. investigated the effects of the cooperation between dietary supplementation with BBs and fat diets on mice subjected to manganese (Mn)-induced neurotoxicity [47]. BB supplementation protected mice with low-fat and normal-fat diets; in contrast, the effect was negligible in HFD mice, suggesting that BBs had their greatest benefits for age-related neurodegenerative disorders when consumed with a low-fat diet. In addition, the effects of BBs in enhancing signaling, cognitive impairment, and declining behavioral decrements were found in an Alzheimer’s disease model [48,49]. Four-month-old APP+PS1 transgenic mice fed BB aqueous extract (18.6 g per kilogram of diet) showed no changes in amyloid beta burden at 12 months old. However, BBs did show effects on enhancing the memory-associated neuronal signaling pathway and modifying neutral sphingomyelin-specific phospholipase C activity, which are protective mechanisms in Alzheimer’s disease [48]. Researchers also found a connection between the expression of proteins involved in cognitive dysfunction and the improvement of this impairment via an analysis of proteomic profiles of the hippocampus of APP/PS1 transgenic mice after feeding them with BB extract (daily dose of 150 mg/kg·bw for 16 weeks) [49]. Scientists have continually updated their research on the benefits of BBs in neuroprotection, hoping to slow the pace of the brain ageing process and related diseases. Bioavailability, the levels of valuable BB compounds appearing in the brain, and the kind of supplemental form that provides the best efficiency, are considered top areas of research.

## 3. Bioavailability and Different Forms of BB Consumption from Animal to Human Studies

Different doses and types of BB intake could provide different amounts of ACNs, leading to the reputation of BBs as having one of the greatest antioxidant effects among fruits and vegetables. ACN interventions can take the forms of smoothies and juices to facilitate administration. Despite its health potential, ACNs in general, including ACNs extracted from BBs, are unstable; they are easily degraded under various conditions, such as high pH, light, enzymes, and oxygen [55,56]. Moreover, ACN levels are strongly affected by food processing and storage conditions [57,58]. The bioavailability of ACNs hence has been investigated to discover if it hinders the widely known medical effectiveness of ACNs. While ACNs are not significantly affected by in vitro gastric digestion with regard to composition and antioxidant properties, a loss of over 40% of total ACNs and nearly 30% of antioxidant activity has been observed during intestinal digestion [59]. Liu et al. found that the high absorption efficiency of ACNs is influenced by their glycoside structure [59]. In in vivo studies, ACNs are rapidly absorbed and eliminated, resulting in low plasma concentrations. No ACNs were detected in the plasma and urine of fasted pigs following BB supplementation for 4 weeks [60]. However, the study showed that intact ACNs were distributed in tissues including the blood-brain barrier, liver, eye, cortex, and cerebellum of the pigs. In an in vivo study of rats fed BBs for 4 or 8 weeks, ACNs were not detected in plasma or in the liver and brain but were significantly increased in urine [61]. However, metabolites of ACNs were found in plasma, urine, feces, and tissues, among which the hippuric acid detected in urine and likely produced in the liver via a conjugation of glycine with aromatic phenolic acids was highest, indicating its potential use as a biomarker of ACN absorption [61]. Of note, the bioavailability and metabolism of ACNs depend on several factors, including dose, food and drink intake, age, and sex of the subjects and the methods used to analyze ACNs in these studies. Because ACNs are mainly administered orally, the bioavailability of ACNs is affected by factors common to other substances taken by oral administration, including movement through biological membranes, pH, food, digestive enzymes, motility, and permeability of the gastrointestinal tract (GIT), and all other factors involved in absorption, distribution, metabolism, and elimination. For instance, the solubilization and absorption of ACNs may be enhanced in the presence of lipophilic food or ethanol [62,63]. Any compound consumed from food during a meal should be considered for its possibility of interacting with ACNs, as ACNs interact with proteins, as reported in [64]. The bioavailability of ACNs is also affected by the microbiota of the human gut, whose characteristics can be driven by dietary habits. Indeed, the effect mainly occurs on metabolites of ACNs rather than on the ACNs themselves [65]. Hence, the total ACN bioavailability should consider the complete contribution of all forms of ACNs that cross physiological barriers. Researchers have shown that the long-term consumption of ACNs is likely to facilitate their transport [62], which is encouraging for dietary habits involving ACN-rich foods such as BBs. In this context, the BB preparation, for example, smoothies or juices, that best exerts the health benefits of the fruit should be investigated. In a randomized, cross-over, bioavailability study by Kuntz et al., subjects received 0.33 L of ACN-rich grape/BB juice (841 mg ACN/L) and smoothies (983 mg ACN/L) [66]. Interestingly, little difference between BB juice and smoothie was found from plasma pharmacokinetics and the recovery of urinary metabolites of ACN, except for the greater bioavailability of the phenolic acid 3,4-DHB from juice than from smoothie. Other factors related to the food matrix, such as sugar and fat content, may be considered in deciding which form of intake would be better to consume.

In human studies investigating the effects of BBs on neuroprotection and other diseases/symptoms of neurological complexities, researchers have commonly used BB juice, powder, extract, or whole fruit. Whyte et al. performed a randomized, double blinded, placebo-controlled study to investigate and compare the effects of BB powder and BB extract on episodic and working memory, executive function, mood, and cardiovascular health over a 6-month period [67]. Their results showed that the participants who were assigned BB extract showed an improvement of episodic memory in delayed word recognition and visuospatial Corsi Block tests as well as a lower systolic blood pressure after 3 months [67]. In a trial by Bowtell and coworkers involving 26 healthy subjects between 65 and 77 years of age, half of the subjects who received 30 mL of BB concentrate, equivalent to 387 mg anthocyanidins (and 230 g of whole BBs), each day for 12 weeks, demonstrated significantly increased brain activity and grey matter perfusion in the parietal and occipital lobes [68]. Thus, anthocyanin-rich BB supplementation has been suggested to improve blood flow to the brain and working memory and activate brain areas associated with cognitive function in older adults [68]. In another randomized, double-blind, placebo-controlled trial, older men and women were administered daily fish oil, BB powder, or both over 24 weeks [69]. BB powder, which was equivalent to one cup of whole BB fruit (25 g, daily dose) taken at 12.5 g per packet twice a day with breakfast and dinner, affected cognitive enhancement through improved memory discrimination [69]. BB powder can also be added to yogurt and skim milk-based smoothies [70] or suspended in water as a drink [9]. One of the first studies on the benefits of BBs on children was an acute intervention of BB drink (15 or 30 g of freeze-dried BB powder) after 2–6 h, which could enhance executive function, short-term memory, and mood in 7–10-year-old children [71]. Specifically, the effects of BB on cognitive improvement were observed by repeating a list of words after 1.15 h, maintaining delayed memory performance for 6 h, and improving accuracy on cognitively demanding incongruent trials in an interference task after 3 h [71]. Cognitive performance was assessed in a dose-response manner, in which the best performance was achieved with a 30 g BB drink [71]. In addition, the time points when it could demonstrate a positive effect were critical, i.e., such positive performances were not observed at other time points. Another study investigating this improvement in more detail [72] showed that a BB product, equivalent to 240 g or 1½ cups of fresh BBs, can have an acute effect on cognition in children and was particularly beneficial for critical periods of brain function development. Most recently, the cognitive effects of acute BB ingestion in this group of children continued to be reported with further findings on extended memory (after 75 min) and executive function task batteries (after 3 h) [73]. This study showed contrasting results with previous research where more cognitively demanding tasks substantially demonstrated improved accuracy and reaction times, suggesting further investigations on determining the association between brain areas and corresponding cognitive functions as well as their mechanism of action [73]. Meanwhile, the improvement of world list recall in adults was recorded after 12 weeks of consumption of BB juice [74]. In an attempt to exploit the benefits of BB consumption in improving the memory of older adults, a group of researchers from the USA and Canada provided BB juice to seniors over 70 years old with a mean educational level of 15.6 ± 1.5 years [74]. Daily consumption of wild BB juice by 9 older adults resulted in improved paired associate learning and word list recall and likely reduced depressive symptoms and glucose levels [74]. This kind of supplementation in the middle term could benefit neurocognitive impairment and establish evidence to further perform human trials to obtain more details on understanding the ability of BBs to prevent cognitive ageing and its neuronal mechanisms [74]. Another research group found that daily consumption of 2 cups of BBs for 6 weeks could improve gait control during a challenging dual-task gait test among adults over 60 years old [75]. Interestingly, in the control group of this study, participants received carrot juice, another food containing ACNs, indicating the greater benefits of BB intake. In contrast, Miller et al. recently reported no improvement in gait or balance when conducting a randomized, double-blind, placebo-controlled trial for 90 days in healthy seniors aged 60 to 75 who received either freeze-dried BB (24 g/day, equivalent to 1 cup of fresh BBs) or a placebo [76]. However, subjects in the BB group had significantly fewer repetition errors in the California Verbal Learning test and reduced switching costs on a task-switching test, suggesting that a BB diet could relatively improve cognition in old adults [76]. Table 3 demonstrates the specifications of these human studies, including the supplementation forms and daily quantities of BBs (with the equivalent amount of ACNs) and the beneficial effects of these ingestions on neuronal functions.

In these human studies, when the subjects were given a task to test cognitive performance, brain-derived neurotrophic factor (BDNF) was considered a critical factor for elucidating cognitive improvement, including outcomes of short- and long-term memory learning [77,78]. For instance, an interference task such as the modified flanker task, which is sensitive to acute exercise (e.g., walking), is associated with increased BDNF and cerebral blood flow (CBF) [71,79]. BDNF levels have been found to be maintained or upregulated after consumption of an anthocyanin-rich diet, inducing strong memory effects on encoding and recalling word lists during recognition tasks [71,80]. In addition, elevated CBF was found following consumption of flavanol drinks, with a peak response after 2 h of consumption and a return to baseline after 6 hrs [81,82]. Such an increase in CBF, leading to a stimulation of neuronal function, is closely correlated with benefits on memory tasks associated with the hippocampus [82]. Flavanol supplementation has been found to improve hippocampal function and cerebral perfusion [82,83]. Anthocyanins were spotted in the hippocampus and neocortex, areas of cognitive function, after BB interventions [50], which identified their susceptibility to being distributed in the hippocampus and involvement in this region’s increased signaling [44]. Another explanation suggested was that the increased CBF would lead to an increase in oxygen [71] that could influence cognitive function and brain activity [84]. In addition, an increased level of CBF will increase glucose transport [85], which affects cognitive function and neuroprotection via enhanced glucose uptake [86]. The greatest memory benefits of BB in cognitive tests were specifically recorded approximately 1–2 h and 6 h after consumption but at no other time, which is in accordance with research on the metabolism of anthocyanin in BB flavonoid intake and its effects on vascular function [87], which in turn clarifies how improved cognition may be associated with increased blood flow. Several findings have been reported regarding the capability of flavonoid compounds to modulate central signaling cascades leading to changes in neurovascular supply and the mechanisms underlying the association between the endothelium and improved vascular function [88,89,90]. Protein kinases (e.g., MAPK, ERK, CREB) are also thought to be involved in learning and memory effects induced by BBs [91,92,93,94], which are also affected by differences in ageing that lead to different levels of memory, signal, and sensitivity to ROS [92,95,96]. These findings support accumulating evidence clarifying the beneficial effects of the BBs on neuronal functions. For example, researchers found that BBs are able to reduce oxidative stress and inflammation, as well as alter cell signaling to improve neuronal communication [88,97,98], which are actions important at certain ages when the brain becomes more vulnerable to oxidative stress and reduces its ability to protect and renew damaged cells. Other possible mechanisms include the ability of BBs to reduce stress signals (NF-ĸB), generate neuroprotective heat shock proteins in response to stress, and function as a buffer against excess calcium [45,99,100,101]. More details of the BB mechanism of action in neuroprotection, neurodegenerative diseases, cognition, and other related brain benefits can be found in several published articles [102,103,104,105,106,107,108,109,110,111,112,113].

Moreover, clinical trials have been conducted to investigate the effects of BBs and their mechanism of action in brain health. Most recently, a trial has been performed to explore the impact of a 6-month daily intake of BB powder (450 or 900 mg) and extract (100 mg) contained in a capsule on cognitive behaviors in seniors aged 65–80 [114]; another trial is shedding light on BB’s mechanism in enhancing cognitive performance through an increased vascular efficiency in adults over 60 years old [115]. The former, a randomized, double-blind, placebo-controlled study, was conducted in subjects who had memory issues and were willing to maintain their normal habits of eating and exercise to maintain a constant body weight during the trial. Primary outcome measures were recorded through correct word recognition, correct recall of sequences of 9 white squares, and combined delayed word recall and word and picture recognition. Secondary outcome measures were also recorded with regard to reaction time for incongruent attention network and Stroop task trials, immediate words recalled, number of correct serial 3 and serial 7 subtractions, Sternberg Task, systolic blood pressure, diastolic blood pressure, heart rate, and positive/negative affect score [114]. The latter study, a 3-month randomized, double-blind, placebo-controlled trial (subjects received drinks of 36 g BB per day in a split dose consumed with meals), evaluated whether routine exercise combined with daily BB consumption affects cognitive function in a manner related to cardiovascular function. In this study, subjects were overweight but had well-controlled blood pressure and normal cognition and could walk independently. The primary outcome is vascular function; secondary measures include 24-hr ambulatory blood pressure, cognitive performance, physical activity, aerobic endurance, hand grip strength, and other outcome measures, such as central arterial pressure, body weight/height, seated blood pressure, verbal memory, executive function, change in processing speed and reaction time, paired associates learning, spatial working/pattern recognition memory, delayed matching to sample, and rapid visual information processing [115].

## 4. Technology in Fabricating ACN-rich BBs into Delivery Systems

**Figure 2:**
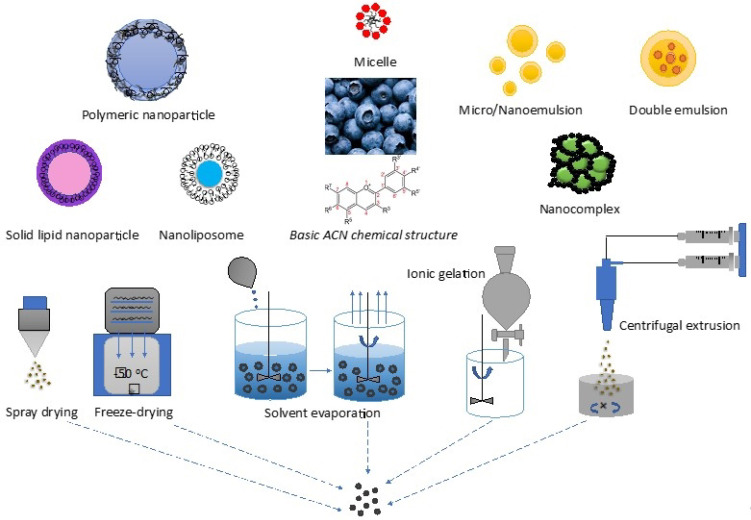
Microencapsulation/nanoencapsulation of BB ACNs to protect and improve their bioavailability. (Top) Different ACN delivery systems/vehicles. (Bottom) Typical techniques commonly used in encapsulation.

In the application of BBs to delivery systems, most studies have focused on BB extracts and on their ACNs. Unfortunately, ACNs have low bioavailability, as mentioned in the previous section. Thus, researchers have worked on strategies that can protect ACNs from degradation and improve their bioavailability. A computer-regulated gastrointestinal model (TNO^TM^, TIM-1) displayed the outcome of using an edible protein-based matrix to deliver BB ACNs; that is, the ACNs were better protected from degradation in the GIT than ACNs in common BB juices [116]. This result suggests a useful strategy to formulate a colon delivery system for ACNs. In addition, researchers have developed other techniques to protect these compounds and enhance their stability to promote their pharmacological activity on sites of action. Micro/nanoencapsulation is an effective technology for facilitating the protection and delivery of BB extracts/ACNs. Various methods for encapsulation include spray-drying, freeze-drying, spray-cooling, fluid-bed coating, extrusion spheronization, centrifugal extrusion, hot melt coating, ionic gelation, solvent evaporation extraction, and coacervation, as well as chemical methods, including polycondensation, polymerization, and interfacial crosslinking (Figure 2) [117,118]. Ionic gelation has emerged as one of the best methods for preparing encapsulations due to its simplicity, compatibility, non-toxicity, and convenience. In addition, the selection of suitable materials for encapsulating unstable compounds is extremely important. Commonly, materials that have the ability to delay the release of active ingredients have been preferably selected. Micro/nanoencapsulation methods indeed control the release of active ingredients at determined times and locations through multiple interactions between these ingredients and polymers that are used to form the walls of the capsules.

A microencapsulation process performed by Wang et al. was developed to improve the encapsulation efficiency and stability of BB ACN extract by using chitosan as the wall material and cellulose nanocrystals (CNCs) as a macroion crosslinking agent through the ionic gelation method [119]. The study showed that the incorporation of CNCs into chitosan microcapsules stabilized BB loading at pH 7.4, demonstrated by the lowered release of ACN, and the CNCs played the role of both a crosslinking agent and a filler to generate a rigid and stable chitosan matrix [119]. This study hence indicates that a combination of two or more components in the wall materials would be an ideal formulation for microcapsules for a high encapsulation efficiency and stability of unstable polyphenols extracted from BBs or other fruits. In a later study, Cai et al. encapsulated BB ACNs with different ratios of carboxymethyl starch (CMS)/xanthan gum (XG) combinations by freeze-drying [120]. As a result, the resulting microencapsulation increased the thermal stability and encapsulation efficiency of the ACN loading process due to the crosslinking interactions between the polymer combinations and ACNs. Interestingly, the stability of the ACNs was increased over 70% after 30 days of storage at 37 °C, and the release of ACNs was mostly delayed in the stomach but on-time in the intestine [120]. Of note, the release of ACN was inversely proportional to the XG content and was dependent on the pH conditions that determine the dissociation of the carboxymethyl group of the CMS and the interactions between the CMS and XG, which reduced the expansion of the microcapsules and formed an XG hydration gel that prevented the digestion of the CMS-based microcapsules by the enzymes of the gastrointestinal (GI) organs [120]. Meanwhile, a research group from Brazil microencapsulated BB ACNs in wall materials composed of maltodextrin DE20 and hi-maize by spray-drying to evaluate the characterization, stability, and delivery of the compounds under simulated GI conditions [121]. This study demonstrated that different wall materials and inlet temperatures in the spray-drying process could determine the encapsulation efficiency, and the degradation and half-life of the microcapsules during storage were largely dependent on the ratio of the components and temperature used to generate the microcapsules, which were found to improve delivery of the compound under GI conditions [121].

To investigate the importance of the selection of wall materials in microencapsulation for facilitating the release and preventing the degradation of ACNs in GI conditions, Wu et al. used four different wall materials, namely, gelatine, soy protein isolate, maltodextrin, and arabic gum [122]. In this study, they also explored the effect of wall materials on the modulation of gut microbiota under simulated GI digestion and colonic fermentation conditions [122]. An appropriate selection of wall materials could determine the effective encapsulation, release, and colonic accessibility of ACNs. Soy protein isolate was demonstrated as the best wall material in terms of the delayed release of ACNs and promoting gut health to decrease the related disease risks [122]. The cyclodextrin complex is a special case in which the active ingredient molecules are included in the cavity of the cyclodextrin structures by van der Waals forces, hydrophobic interactions, or hydrogen bonds to form complexes [123]. Xu et al. improved the stability of BB ACNs by microencapsulating them with β-cyclodextrin-whey protein-arabic gum via a spray-drying method, achieving a high encapsulation efficiency of 95% [124]. In addition, microemulsions have also been used to enhance ACN stability and bioavailability. Chen et al. developed a pseudo-ternary phase diagram composed of oil, surfactant, and cosurfactant (isopropyl myristate, Tween 80/Span 80 and ethanol, respectively) to encapsulate BB ACNs [125]. This water-in-oil microemulsion helped ACNs retain more stability against temperature and light than ACNs in solution. Although microencapsulation shows advantages comparable to other delivery systems, the following example would give researchers more evidence to continue with investigations in the field. Flores et al. spray-dried BB extracts with whey protein isolate for microencapsulation and investigated the release behaviors of the encapsulated BB extracts (W) from the released monomeric anthocyanins and phenolics as well as ferric reduced antioxidant activity compared with that of encapsulated ethanolic pomace extract (P) and freeze-dried juice (F) [126]. Surprisingly, more phenolics were found to be released from the W and P encapsulation types than from the F encapsulation type, and the ACN concentration of W decreased while remaining constant in the P and F types. In addition, the antioxidant activity of F was also higher than that of W, which itself was higher than that of P. Disadvantages of microencapsulation have been reported, including insufficient delivery of the loading ACNs to their absorption sites, use of excessive reagents, high costs, moderate yields, and existing problems associated with solubility, particle size, and flow rate [127].

Nanoencapsulation is emerging as an alternative method for encapsulating unstable compounds and is predicted to contribute fruitful outcomes to this field, providing a promising delivery strategy to enhance drug bioavailability in healthcare areas such as neurodegenerative diseases and brain tumors [128,129,130,131]. This approach is also expected to result in targeted drug delivery systems that can protect and precisely deliver such bioactive compounds to desired sites [132,133,134,135]. Nanocapsules can be fabricated under formations such as polymeric nanoparticles, nanocomplexes, solid lipid nanoparticles, nanoliposomes, and micelles (Figure 2) [128,134,136,137]. Subsequent studies have investigated the use of nanoencapsulation for ACNs. Ge et al. prepared nanocomplexes composed of chitosan hydrochloride incorporated with carboxymethyl chitosan and β-lactoglobulin as the wall material for loading ACNs as the cargo [138]. The nanocomplexes were capable of providing sustained release behaviors, improving the stability and bioavailability of ACNs in GI conditions. Chondroitin sulfate was used to form nanocomplexes with ACNs and was then embedded in a kappa-carrageenan hydrogel to enhance the stability of ACNs [139]. This is an example of a versatile approach to achieve different forms other than micro/nanostructures to encapsulate ACNs for assessing the delivery and stability of ACNs. Guo et al. encapsulated BB extract in alginate-pectin hydrogel particles (with a diameter of nearly 3 mm, although different sizes and shapes depend on the distance of the dropping solutions) [140]. The encapsulation significantly minimized the photodegradation of ACNs and was best achieved with spherical hydrogel particles through an evaluation of the retained ACNs in the particles [140]. In another study, Bamba et al. prepared a double emulsion (water/oil/water) with whey protein isolate, corn oil, polyglycerol, and ethanol via homogenization (10 min at 10,000 rpm and then 6000 rpm for 15 min) and microfluidization at 50 MPa [141]. The study showed a high encapsulation efficiency of BB polyphenols and ACNs with an average oil droplet diameter below 400 nm, a relatively low PDI (below 0.25), and a high negative zeta potential (approximately −40 mV), predicting the stability of the emulsion system. Nanoliposomes are another potential delivery system for unstable compounds due to their protective structure composed of an aqueous core and bilayer membrane. These vesicles have been used to enhance the stability of ACN-rich natural extracts from berries via the fabrication of lecithin-based chitosan-coated nanoliposomes (by high shear dispersion (9500 rpm/10 min) and high pressure homogenization (25,000 psi, 5 passes)) and, interestingly, particularly maximize the stability of ACN when incorporated with dark chocolates [142] and soybean lecithin nanoliposomes (by the thin lipid film hydration technique followed by freeze and thaw processes) [143]. Nanoliposomes themselves are stabilized by the presence of antioxidants such as ACNs and biopolymer-based coatings (for example, layer-by-layer chitosan/pectin-coated liposomes) [144,145]. Although it has yet not been applied to ACNs, orally disintegrating films composed of gelatine/starch were used as potential carriers of vitamin C, which, similar to ACNs, is a kind of antioxidant compound that is likely to degrade under light [146]. This research is certain to promote further investigations into strategies to protect and enhance the bioavailability of other unstable substances, such as ACNs.

## 5. Conclusions

Many promising outcomes are revealed in support of the benefits of BB interventions in neuroprotection. However, frequent supplementation of BBs over long periods and more complex, sensitive tests are needed to comprehensively assess the benefits of BBs for healthy brain functions. In addition, it remains to be determined whether BB interventions should be performed at younger ages or when supplementation should be administered to achieve much greater performance of cognitive behaviors, as interventions at earlier epochs of cognitive ageing might produce greater or more rapid performance enhancement. Further pharmacological/pharmacokinetic studies should investigate the effect of BB consumption types (whole fruit, powder with meals, drinks—simple water or smoothies, or encapsulated forms) on brain health benefits and thus suggest the best way to introduce BB interventions into the body. Of note, eligibility criteria should be carefully considered for the recruitment of subjects in human studies to select relevant information as inclusion criteria and exclude factors that may affect the outcome, such as current use of alternative medicine, medical history, exercise habits, and habits of daily food intake. In addition to the whole fruit, BB leaves have also been revealed to have potential brain health benefits due to their even higher polyphenolic content and antioxidant properties. These explorations would promote further studies on BB leaves regarding favorable edible supplementation forms and the manner in which maximum absorption and bioavailability can be achieved to broaden the applications of BBs in providing healthcare for neurological-related complexities.

## Figures and Tables

**Table 1:** Potential in vitro effects of Blueberries (BBs) on brain health protection.

Consumption Type	Key Results	References
BB extracts	Lowering the gene expression of _Mus_IL-1b, _Mus_NLRP3, _Mus_Caspase-1, _Mus_ASC, _Mus_TNF-a, _Mus_IL-6, and _Mus_iNOS and the protein expression of NLRP3 and Caspase-1	[30]
BB fruit or leaf extracts	Inhibiting cell death and decreased inflammatory conditions	[31,32]
BB extract	Protective ability of BB ACNs for microglia undergoing inflammatory stress	[33]
BB extracts	Protecting microglia from inflammation-induced stress signaling	[34]
BB extracts	Human microvascular endothelial cells were protected from TNFα-induced damage	[35]

**Table 2:** In vivo studies of BBs on brain health protection.

Consumption Type/Animal Models	Key Results	References
BB supplementation/Fischer 344 rats	A decrease in the activation of microglia	[42]
Dried aqueous extract/Fischer 344 rats	Reversing age-related deficits in several neuronal signaling pathways and behaviors	[43]
BB extract/Fischer 344 rats	Increasing hippocampal plasticity parameters, IGF-1 and IGF-1R levels	[44]
BB diet/Sprague-Dawley rats	Anti-inflammatory activity in post-traumatic stress disorder	[45]
Freeze-dried BBs/C57Bl/6 mice	Fewer microglia and increased neuroplasticity in the brains of high-fat diet mice than in those of high-fat diet mice who did not consume BBs and of low-fat diet mice	[46]
Dietary supplementation with BBs and fat diets/C57BL6/J mice	Greatest benefits for age-related neurodegenerative disorders when consumed with a low-fat diet	[47]
BB extract/a mixture of C57BL/6, DBA, SJL and Swiss Webster mice	Enhancing the memory-associated neuronal signaling pathway and modifying neutral sphingomyelin-specific phospholipase C activity	[48]
BB extract/APP/PS1 transgenic mice	A connection between the expression of proteins involved in cognitive dysfunction and the improvement of this impairment	[49]

**Table 3:** Clinical trials on the effects of blueberry consumption on brain health.

Consumption Type	Quantity (Daily Intake)	Equivalent Anthocyanins (ACNs)	Age Group	Number of Participants	Effects	Reference
Extract vs. powder (encapsulated in transparent capsules)	Extract: 100 mg Powder: 500 or 1000 mg	7 mg 1.35 or 2.7 mg	65–80	122	3-month intervention: extract facilitated better episodic memory performance (improved cardiovascular function over 6 months)	[67]
Concentrate (30 mL, diluted to 240 mL with water)	230 g	387 mg	65–77	26	12-week ingestion: improved cognitive function and working memory	[68]
Powder (whole frozen BBs freeze-dried and powdered to 20 mesh/consumed with morning and evening meals)	25 g (12.5 g per packet, twice a day)	269 mg	62–80	94	24-week intervention: cognitive enhancement	[69]
Powder (freeze-dried/mixed with 30 mL of low-energy fruit squash, Rocks^®^ UK and 170 mL of water)	15 or 30 g (equivalent to ~ 120 or 240 g fresh BBs)	127 or 253 mg	7–10	24	30 g dose showed the better effect. Consumption improved cognitive performance and immediate recall 1.15 h following the intervention; improved word recognition and accuracy on cognitively demanding incongruent trials in the interference task 3 h following the intervention; improved epi- sodic memory at 75 min and executive function 3 h post-consumption.	[71,72,73]
Juice	Individuals 54–64 kg: 444 mL/day; 65–76 kg: 532 mL/day; 77–91 kg: 621 mL/day	0.428 g 0.512 g 0.598 g	76.2 ± 5.2	9	Moderate-term BB supplementation provided a preventive effect on neurocognitive function.	[74]
Fruit (frozen BBs)	~0.46 kg (2 cups daily)	N/A	61–81	20	6-week consumption: Positive impacts on age-related declines in functional mobility.	[75]
Freeze-dried BBs	24 g	N/A	60–75	40	3-month intervention: improved cognitive function (significantly fewer repetition errors in verbal learning tests and reduced switching costs on a task-switching test) without improvement in gait or balance.	[76]

## Data Availability

Not applicable
